# Study on the Effect of Metal Mesh on Pulsed Eddy-Current Testing of Corrosion under Insulation Using an Early-Phase Signal Feature

**DOI:** 10.3390/ma16041451

**Published:** 2023-02-09

**Authors:** Hanqing Chen, Zhiyuan Xu, Zhen Zhou, Junqi Jin, Zihua Hu

**Affiliations:** School of Mechanical Engineering and Mechanics, Xiangtan University, Xiangtan 411105, China

**Keywords:** pulsed eddy-current testing, insulated piping, corrosion under insulation, metal mesh, probe footprint

## Abstract

Corrosion under insulation (CUI) is a major threat to the structural integrity of insulated pipes and vessels. Pulsed eddy-current testing (PECT) is well known in the industry for detecting CUI, but its readings can be easily influenced by nearby conductive objects, including the insulation supporting metal mesh. As a sequel to our previous study, this paper focuses on the surface distribution of eddy currents at the time of the turning off of the driving voltage instead of examining the overall process of eddy current diffusion. Based on the fact that CUI takes place on the outside of the insulated specimen, the probe footprint was calculated only on the specimen surface. The corrosion depth was regarded as an increment to the probe lift-off, whose information was carried in the early PECT signal. Finite element simulations were performed to facilitate the calculation of the probe footprint and predict the signal behavior. The peak value, which appeared in the early phase of the differential PECT signal, was found to be well correlated with the corrosion depth. Further studies revealed that the mild steel mesh could result in the enlargement of the probe footprint and a decrease in the change rate of the peak value in relation to the corrosion depth. Finally, experiments were conducted to verify the simulation results. The presented findings are consistent with the previously reported results and provide a potential alternative to evaluate CUI in specific scenarios where the insulation has a fixed and uniform thickness.

## 1. Introduction

Corrosion under insulation (CUI) is a form of external corrosion that occurs on the underlying metal beneath the thermal-insulated coating [1]. It stems from the accumulating electrochemical corrosion due to ingress of water/moisture into the insulation. In power generation, refining and petrochemical plants where the majority of the piping is made of ferromagnetic materials (usually carbon steel), CUI mostly manifests as general corrosion. This type of corrosion challenges the structural integrity and long-term safe operation of the insulated equipment, and usually accounts for a major proportion of the repair and maintenance costs. Unfortunately, the outer insulation conceals the onset of corrosion and hinders the deployment of traditional nondestructive testing (NDT) techniques, such as visual inspection and ultrasound testing, unless the insulation is removed [2]. For cost and efficiency reasons, it is highly desirable to apply an NDT method with the ability to inspect CUI across the insulation coating.

Until now, various NDT methods have been reported in the literature to address the challenge of CUI inspection without removing the insulation, including infrared thermography [3], long-range guided wave ultrasonic testing [4], radiography [5], neutron backscatter [6], optical fiber-based water sensing [7], capacitive imaging [8], and pulsed eddy-current testing (PECT) [2,9,10,11,12]. Among them, PECT has seen steady development and increased applications in recent years. It utilizes a pulsed signal to excite the eddy current rather than the more common sinusoidal signal, which broadens the range of frequencies in the signal and thus, greatly improves the penetration of the signal. Compared with ultrasonic testing and radiography, PECT inspection for CUI does not require coupling agents and avoids the concern of radiation exposure. Therefore, it is often used as a fast screening tool to identify large areas with wall loss, and if necessary, a follow-up ultrasonic test with partial insulation removal is performed to ascertain the remaining wall thickness. The main limitation of PECT inspection for CUI is that the measured steel thickness is averaged over a large area of the test specimen, called the footprint. Furthermore, in actual application conditions, the pipeline is fully insulated, making the exact absolute thickness at any specific location rarely available, and therefore, PECT measurements are usually relative wall thickness readings. One reference spot is selected and assigned a thickness of 100%; then, the thickness at other locations will be presented as a percentage of the thickness at the reference spot, usually in a color-coded C-scan grid [12].

The footprint is considered as an important parameter that determines the sizing performance of the probe. As a rule of thumb, if the corroded area is comparable or larger than the footprint, the remaining wall thickness of the CUI site will be correctly determined; however, if the corrosion is smaller than the footprint, then it will probably be undersized or even be undetected [2,12]. Unfortunately, the footprint size is not a concrete parameter, as are the probe structural parameters. It describes the specimen’s active area where induced eddy currents are concentrated [13]. For a typically used cylindrical probe coil, its footprint is in a circular shape. In empirical analysis, the diameter of the footprint is 1.5× the sum of the lift-off (insulation, jacket, coating thickness) and the wall thickness, with a minimum of 25 mm [14]. However, several other different definitions or calculation methods were found in the literature, which indicates the challenge of providing a general definition for the probe footprint in various application scenarios. Tremblay et al. defined the probe footprint as the full width at half (−6 dB) the maximum of the response detected by the probe, which can be approximated by the formula, FP ≈ 0.65 × *L*_0_ + FP_0_, where *L*_0_ is the lift-off and FP_0_ is the footprint at a lift-off of zero [15]. In their study, the probe footprint was used to determine the optimal grid resolution for proper detection, ensuring a 50% signal overlap between each point on the grid map. Cheng et al. defined the footprint as the area within which the surface eddy current density is above 30% of the maximum surface eddy current density [16]. They used two coils with currents flowing in opposite directions from the transmitter, and found that the footprint size can be reduced, to some extent, at the cost of reducing the eddy current density. Chen et al. proposed an analytical method for calculating the probe footprint based on the equivalent model of the eddy current field in the ferromagnetic plate. It was pointed out that the radial coordinate corresponding to the function value of the radial variation curve of the contribution function decreases to half of the peak value, which can be used as the radius to measure the boundary of the footprint area of the probe [17].

The above studies regarding probe footprint have provided guidance for certain PECT applications in the aspects of probe selection, scan grid division, result interpretation, etc., but were not comprehensive enough, since many factors existing in the field could affect the eddy current distribution and thus the probe footprint. One of these factors is the metal mesh, which is installed in the loose-filled insulation layer (e.g., rock wool and asbestos) to support insulation and help prevent gravity from pulling the insulation out of place. The metal mesh is a metal product with a series of holes made of mild steel, stainless steel, or aluminum wires using a welding or weaving process. Our previous work has studied the effects of the metal mesh on the PECT detection of CUI and found that the mild steel mesh could cause an increase in the probe footprint and a decrease in the decay rate of the probe signal [2]. The decay rate is a late-phase feature of the ferromagnetic material PECT signal presented on a semi-logarithmic scale, which characterizes the fast decay of eddy currents when the downward diffusion stops. It is, therefore, a measure of the remaining wall thickness. In fact, as CUI takes place on the outside of the steel pipe, it alters the distance from the probe to the bottom of the corrosion. In other words, the variation of the corrosion depth causes a change in the probe effective lift-off. It is known that the early phase of the PECT signal contains the information of the probe lift-off [18,19], which inspires us to examine whether the effect of the metal mesh on the PECT detection of CUI could be identified from the early phase of the signal. In light of this, this paper focuses on the analyses of eddy current distribution on the surface of the carbon steel and the change in the early-phase signal features when a metal mesh embedded in the insulation has different geometric and electromagnetic parameters. The effects of the metal mesh on the probe’s footprint size and performance for evaluating the CUI depth were investigated by numerical simulations and then verified by experiments.

## 2. Numerical Simulations

### 2.1. Simulation Model

**Figure 1:**
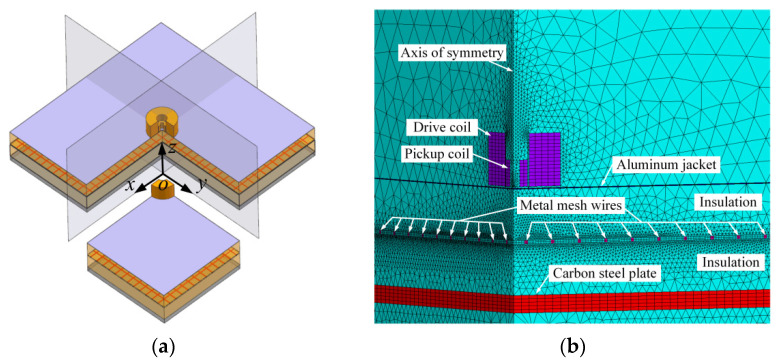
(**a**) Simplified structure of PECT inspection for insulated components, and (**b**) the finite element model.

Numerical simulations allow us to visualize the transient eddy-current field and thus help us understand how the metal mesh affects the field and predict the variation in PECT signals for different metal mesh parameters. Considering that in actual PECT application the probe is much smaller than the pipe under testing, the curved pipe wall can be modeled as a plate for simplification [20]. In this way, the components of the insulated pipe, including the outer jacket, insulation layer, and pipe wall, form a three-layer plate structure. The metal mesh is located in the insulation layer. Figure 1a shows the geometry of the layered structure. As the structure is symmetric in both the *xoz* and *yoz* planes, only a quarter of the entity is modeled to reduce the computational burden. Figure 1b shows the finite element model built in ANSYS 15.0 software. The thickness of the aluminum jacket, insulation, and carbon steel plate are 0.5 mm, 60 mm, and 10 mm, respectively. The entities of regular shape, including the plate, jacket, metal mesh, and probe coils, are discretized with mapped hexahedral elements, while the regions of insulation and air space are meshed with free tetrahedral elements. The aluminum jacket is assigned with an electrical conductivity of 35 MS/m and a relative permeability of 1. The non-conducting insulation is assigned with the air properties. The carbon steel plate is a ferromagnetic material which has a nonlinear permeability dependent on the strength of the applied magnetic field. In PECT practice, the magnetic field works in a condition of low frequency and weak strength; it is generally assumed that the permeability is a constant across the specimen. The conductivity of the carbon steel plate is assumed to be 5 MS/m. Then, simulation signals calculated using various permeability were fitted to the experiment signal, and a good fit was obtained when the permeability was 300 $\mu_{0}$, where $\mu_{0}$ is the permeability of free space equaling $4\pi \times 10^{-7}\;H/m$. The metal mesh under examination is set to have varied material properties and geometric parameters, as shown in Table 1, in which the parameter “position” refers to the distance from the metal mesh to the aluminum jacket.

The probe consists of two co-axially placed pancake coils, of which the outer and inner are used as the drive and pickup coils, respectively. Table 2 lists the coil parameters. The voltage applied to the drive coil, and the induced EMF extracted from the pickup coil, are implemented by coupling a circuit element to the coil elements. The applied voltage source has a repetition frequency of 10 Hz, an amplitude of 4 V, a duty ratio of 0.5, and a leading edge of 1 ms. On the planes of symmetry, the J-normal boundary condition (the current density normal to the area) is set in the areas of conductors. Meanwhile, on the symmetry planes and the outermost air surfaces, the flux parallel condition is applied. After some trials, a time step of 0.5 ms was determined for the transient analysis.

### 2.2. Simulation Results

**Figure 2:**
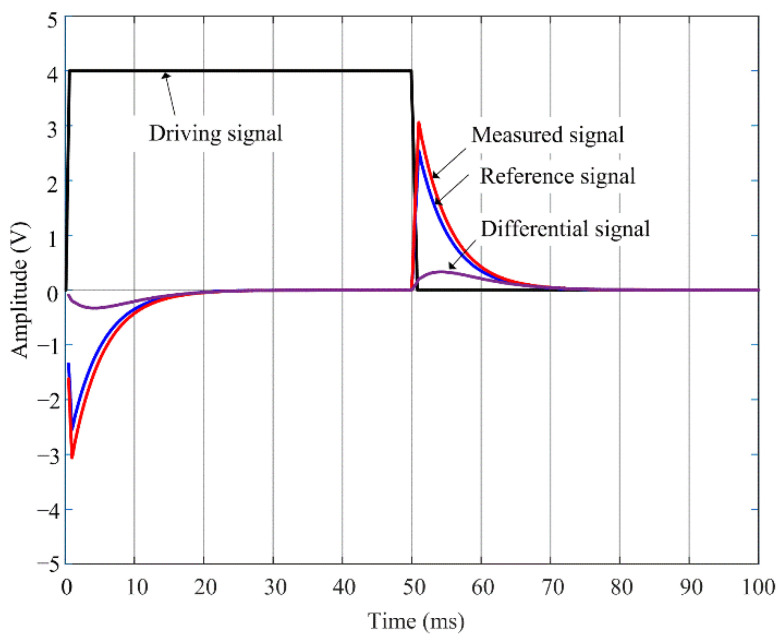
Typical PECT signals presented on a Cartesian plane.

Figure 2 shows the typical PECT signals presented on a Cartesian plane, with a square wave driving voltage, and pickup coil-based induced voltage signals. It can be seen that voltage pulses are induced in the pickup coil at both the rising and falling edges of the driving signal. The two pulse waves are odd symmetric, either of which reflects the time evolution of the PECT signal. Here, the pulse wave at the falling edge is used. The early phase of the pickup signal is dominated by the response to the decay of the signal from the pickup coil, with a high amplitude. The relatively low signal change caused by the induced eddy currents in the specimen would probably be masked. PECT signals acquired on different sites of the specimen might be difficult to distinguish from each other in a large-scale coordinate. For this reason, a reference signal is often used, which is captured from a defect-free or specified area [21]. Then, a differential signal is obtained by subtracting the reference signal from the measured signal. It is clear that the amplitude of the differential signal is much smaller than the original signal amplitude, generally reducing to the order of mV.

**Figure 3:**
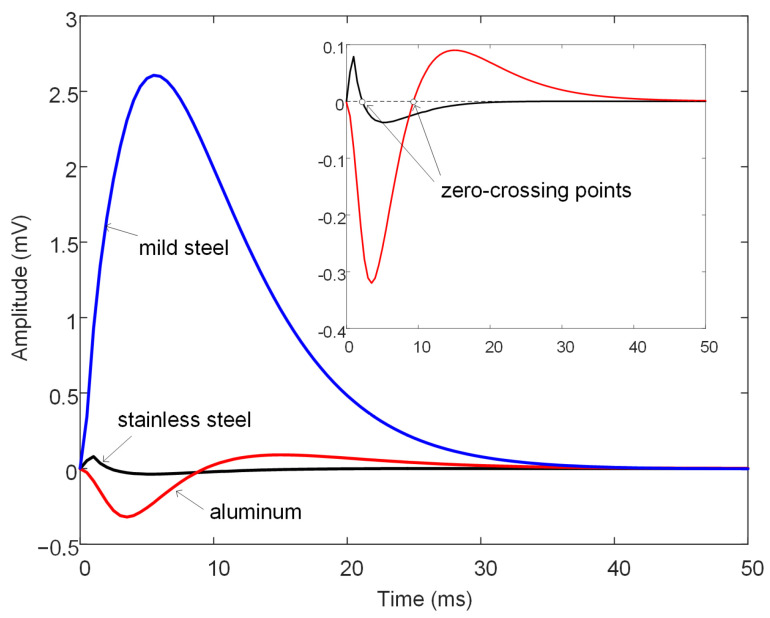
Simulated differential signals for metal meshes of different materials. In the upper right corner is a zoomed-in plot of the low-amplitude signals.

First, three types of metal meshes of stainless steel, aluminum, and mild steel were examined via simulation, with the relative permeabilities being 1, 1, and 200, respectively, and conductivities being 1.35, 21.6, and 10 MS/m, respectively. The geometric parameters, including the wire diameter, hole side length, and position, were equal to 2, 16, and 30 mm, respectively, and were kept consistent for the three types of metal mesh. Figure 3 shows the simulated differential signals in the three cases. In order to highlight the effect caused by the metal mesh, a difference scheme is applied, and the signal calculated from a metal mesh-free model is used as the reference signal. The signal amplitude of the mild steel metal mesh is much larger than the amplitudes of the other two types of meshes, which can be attributed to the ferromagnetic nature of the mild steel. For the non-ferromagnetic stainless steel or aluminum mesh, the secondary magnetic field generated by eddy currents reacts on the pickup coil and induces a voltage opposite to the one induced by the emitting magnetic field; therefore, the differential voltage signal exhibits a zero-crossing phenomenon [22], as shown in the enlargement of the main graph in the upper right corner. The presence of a mild steel mesh not only involves the eddy current effect, but also alters the resistance of the magnetic circuits. It attracts more magnetic flux emitted from the drive coil and hence, amplifies the measured magnetic field. This amplification highly surpasses the reaction of the eddy currents, thereby resulting in a differential signal with a large amplitude.

**Figure 4:**
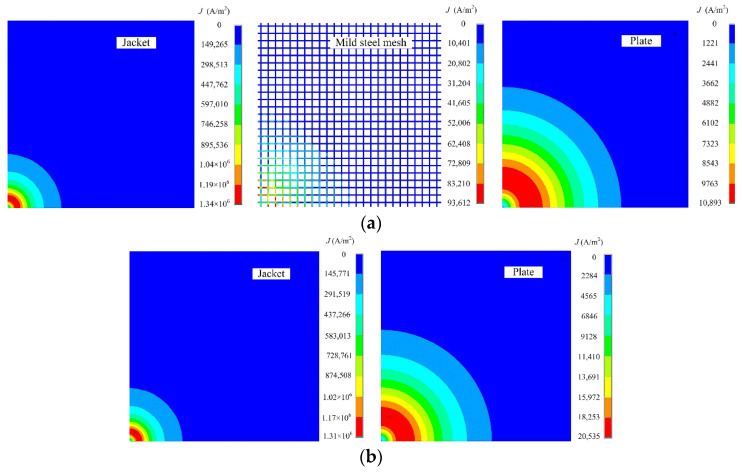
Contour plots of eddy current density in conductive components of the models (**a**) with a mild steel mesh, and (**b**) without any metal mesh, respectively.

Figure 4a shows the distribution of the eddy current in the conductive components of the model at the time point when the driving voltage turns off (i.e., the end of the falling edge). Eddy current is successively induced in the jacket, mild steel mesh, and carbon steel plate. It follows the circular shape of the drive coil and diffuses outward. The ring area in red color on the jacket basically profiles the drive coil bottom, but expands much more on the plate, which indicates that the probe footprint increases with the increase in the conductor-to-probe distance. Meanwhile, according to the color bar, which refers to the eddy current density, the eddy current in the plate is much weaker than that in the jacket. In order to look into the role of the metal mesh, a contrast simulation excluding the metal mesh is performed, and the result is shown in Figure 4b. It is evident that introducing a mild steel mesh might result in two undesirable effects, further increasing the probe footprint size and attenuating eddy currents in the plate.

For quantitative analysis of the effect of the metal mesh, the probe footprint is defined as the plate area within which the surface eddy current density is above 30% of the maximum surface eddy current density [16]. The probe footprint radii are calculated, and the values for the stainless steel, aluminum, and mild steel mesh are 178.7, 173.6, and 200.3 mm, respectively. Compared to the value of 179.5 mm in the metal mesh-free case, the percentage variations of the footprint are −0.44%, −3.27%, and 11.55%, respectively. In the meantime, the maximum eddy current densities on the plate surface in the cases of mesh-free, stainless steel, aluminum, and mild steel are 20,535, 20,530, 20,464, and 10,893 A/m^2^, respectively. Only the mild steel mesh leads to a significant drop (almost 50%) in the eddy current strength. These results confirm the footprint enlargement and eddy current attenuation caused by the mild steel mesh, and the ignorable effect of the stainless steel or aluminum mesh.

**Figure 5:**
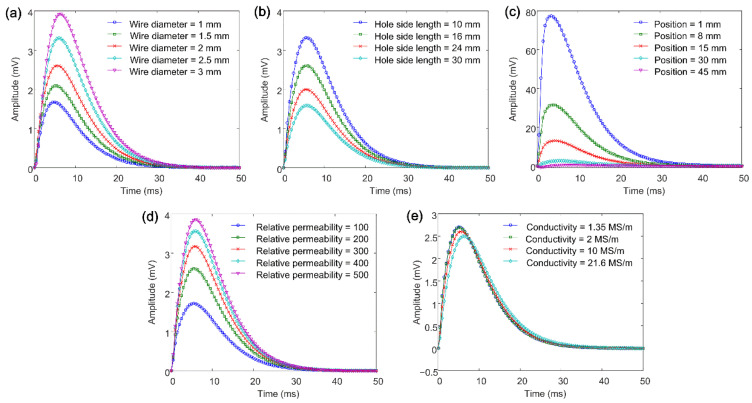
Simulated differential signals for variations in the (**a**) wire diameter, (**b**) hole side length, (**c**) position, (**d**) relative permeability, and (**e**) conductivity of the mild steel mesh.

Therefore, subsequent simulations are focused on the mild steel mesh. Parametric analysis is conducted to find out the main effect factors. The wire diameter, hole side length, position, relative permeability, and conductivity are analyzed separately, and their initial values are 2 mm, 16 mm, 30 mm, 200, and 10 MS/m, respectively. Figure 5 shows the analysis results. As the mild steel mesh’s wire diameter increases, the hole size decreases, the distance to the jacket shrinks, the permeability increases, and the conductivity decreases, the amplitude of the differential signal increases. This also means that the effect of the mild steel mesh becomes stronger. The variation trend caused by the first four factors actually corresponds to the same fact; that is, the more magnetic flux the mild steel mesh attracts from the drive coil, the larger the measured magnetic field. The change in conductivity only affects the induced eddy current, and accordingly, the variation of the signal amplitude is the smallest.

**Figure 6:**
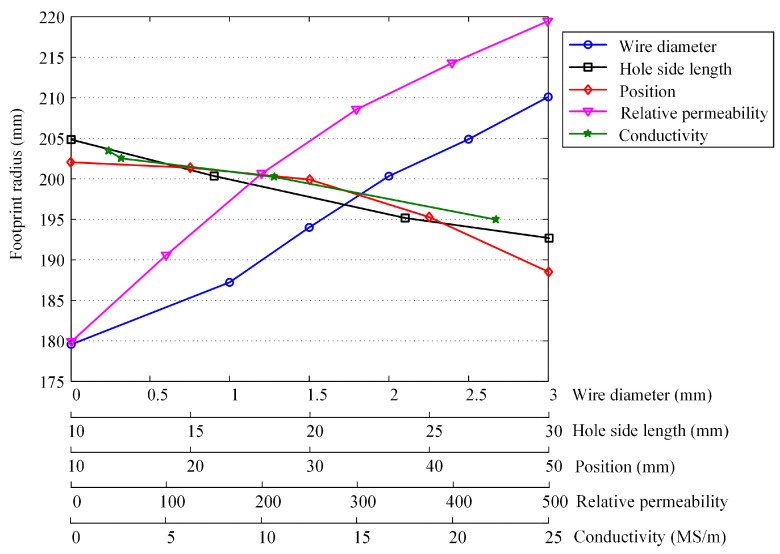
Plots of the probe footprint radius against the mild steel mesh parameters.

The probe footprint is calculated for each simulation model. Figure 6 presents the relationship of the footprint radius versus the mild steel mesh parameters. The footprint radius is positively related to the wire diameter and relative permeability, but has negative relationships with the hole side length, position, and conductivity. From the perspective of the degree of variation of the footprint radius, the relative permeability is the first affecting factor, followed by the wire diameter. The conductivity exhibits the least effect. The footprint radius shows a moderate and almost linear decrease with the increase in the hole side length. The curve of the mesh position descends slowly at the first half, but decreases faster when the mesh is situated lower than the middle of the insulation layer (i.e., the position of 30 mm).

Similar findings are obtained by examining the effect of the mesh parameters on the eddy current density. The magnetic permeability and conductivity are the most and least important factors, respectively. This phenomenon can be explained by the magnetic shielding mechanism. It is the magnetic permeability-related magnetic flux shunt that dominates the shielding of the magnetic field of low frequency, not the eddy current shielding contributed by the electrical conductivity.

**Figure 7:**
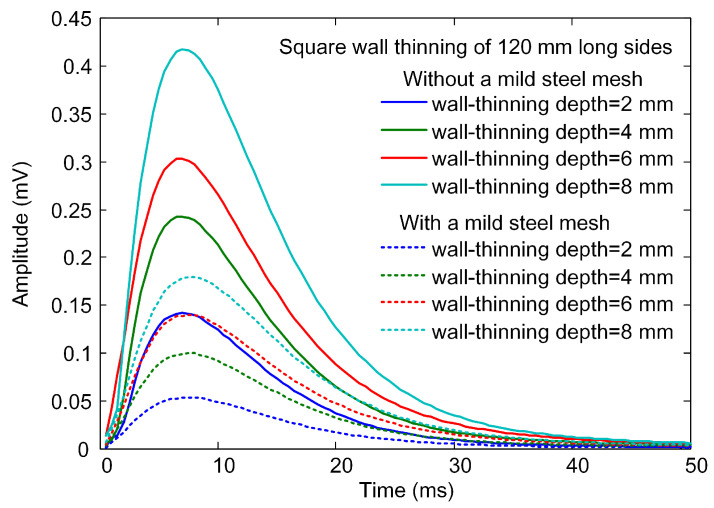
Simulated differential signals for the probe situated above the square wall thinnings having a side length of 120 mm and depths of 2 mm, 4 mm, 6 mm, and 8 mm, respectively.

The increase in footprint radius is accompanied by the attenuation of eddy currents induced in the corroded area. These two effects will make a combined impact on the PECT signal from CUI inspection. Therefore, in the following modeling, a wall thinning of varied depth is created on the upper surface of the carbon steel plate. As the plate is discretized to hexahedral elements, square wall thinnings are formed by replacing the attribute of specified elements with the air attribute. Figure 7 shows the differential signals for the probe positioned above square wall thinnings, having a side length of 120 mm and depths of 2 mm, 4 mm, 6 mm, and 8 mm, respectively. Simulation results for the absence and presence of mild steel mesh are both provided. The signals for the two groups are plotted as solid and dashed curves, respectively, and their referenced signals are obtained from models with a plate of full thickness (10 mm, without thinning). In both groups, the signal amplitude increases with the increase in the wall-thinning depth. From comparison of the two groups, it is clear that the mild steel mesh leads to a significant decrease in the differential signal amplitude.

**Figure 8:**
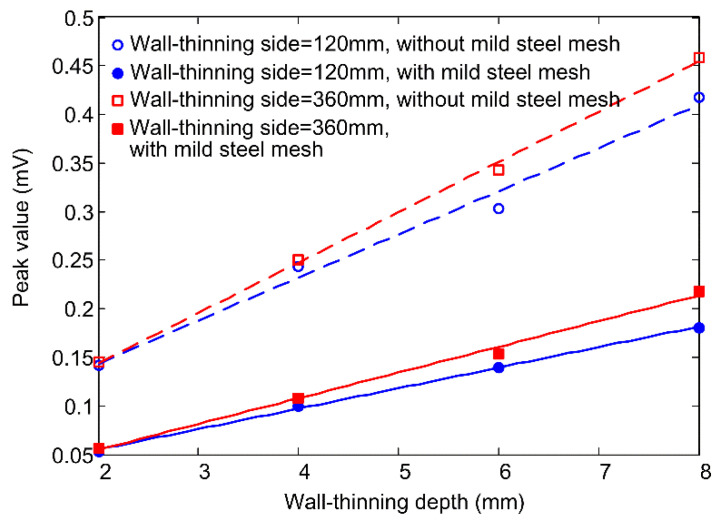
Relationship between the signal peak value and wall-thinning depth for square wall thinnings of 120 mm and 360 mm long sides when the mild steel mesh is absent and present, respectively.

The peak value, arising in the early phase of the differential signal, is used as the feature to evaluate the wall-thinning depth. Figure 8 plots the relationship between the peak value and the wall-thinning depth, when the mild steel mesh is absent and present, respectively. The straight lines represent linear fittings through the data points. As seen from the figure, introducing the mild steel mesh not only makes the peak value decrease, but also slows down the variation in the peak value with the wall-thinning depth. The latter effect is equivalent to reducing the detection sensitivity of the PECT probe, which is attributed to the increase in the probe footprint. For a wall thinning smaller than the probe footprint, it can be inferred that the larger the wall thinning is, the closer the averaged thickness is to the true residual thickness and the larger the corresponding signal differences. To demonstrate this, another group of simulations having a 360 mm long side wall thinning on the plate are carried out. The fitted lines through data points are indeed steeper than those through the 120 mm long side wall thinning data points, which thereby proves the above inference. Furthermore, the phenomenon that the mild steel mesh reduces the detection sensitivity still holds.

## 3. Experiment Validation

### 3.1. PECT System

**Figure 9:**
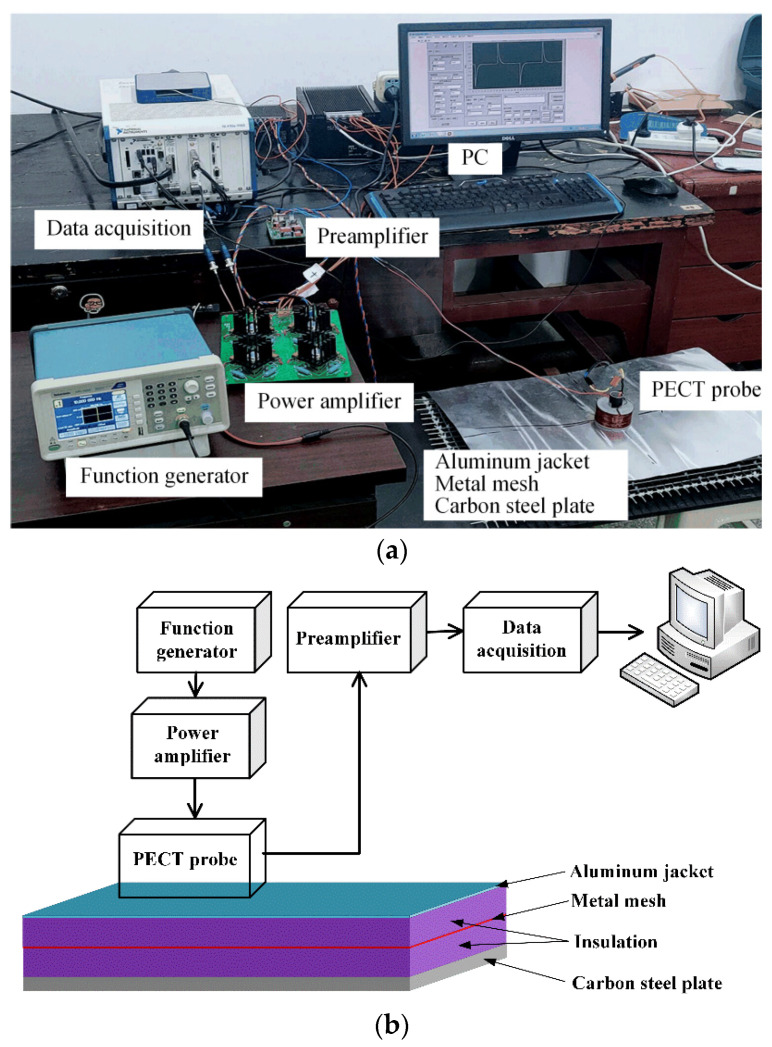
PECT system: (**a**) photo, and (**b**) block diagram.

Figure 9 presents the PECT system built in laboratory, which mainly consists of the PECT probe, function generator, power amplifier, preamplifier, and data acquisition module. The probe’s drive and pickup coils were hand made using 18 and 26 AWG enameled copper wires, respectively, and have the same parameters as those used in the simulation. A 10 Hz square-wave voltage generated by the function generator (AFG1022, Tektronix, Tokyo, Japan) was amplified by a homemade power amplifier and then sent to the drive coil. The pickup coil captured the magnetic field induced by the eddy currents in the sample and then output a voltage signal. This voltage signal is very weak and easily interfered with by the ambient noise. A pre-amplifier, with a cut-off frequency of 1 kHz and a gain of about 50, was thereby developed to preprocess the pickup signal before it was sampled by the data acquisition card (PXIe-4497, National Instruments, Austin, TX, USA). The final data were recorded by an embedded controller (PXIe-8840, National Instruments, Austin, TX, USA), in which a user interface programmed by LabVIEW was developed to set the sampling parameters and display the pickup signal.

**Figure 10:**
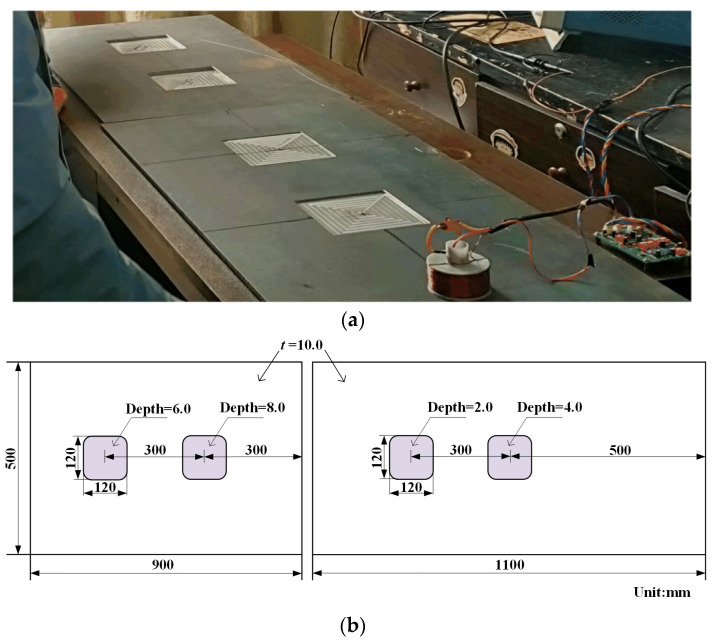
(**a**) Photo and (**b**) dimensions of the sample plates machined with wall thinning defects.

Figure 10 shows the sample plates. Two 10 mm thick Q235 carbon steel plates, manufactured in the same lot, were used. Four flat-bottom square wall-thinning defects measuring 120 mm × 120 mm and with depths of 2 mm, 4 mm, 6 mm, and 8 mm, respectively, were machined on the upper surfaces of the plates. A defect-free zone with the nominal thickness was reserved on the right. The distance between two adjacent defects was 300 mm, which is considered far enough away to prevent inter-defect interference, since the probe footprint radius was determined to 200.3 mm. A metal mesh welded with Q195 mild steel wire of 2 mm diameter was placed above the Q235 plate. It has square holes of 16 mm side length. An aluminum sheet of 0.5 mm was used as the topmost jacket. Some foam plastic shims were stacked onto and under the Q195 mesh to form the 30 mm thick insulation layers. The probe was set on the jacket, making the inner drive and pickup coils 1 mm lifted to the jacket, as the probe has a housing of 1 mm thickness.

### 3.2. Experimental Results

**Figure 11:**
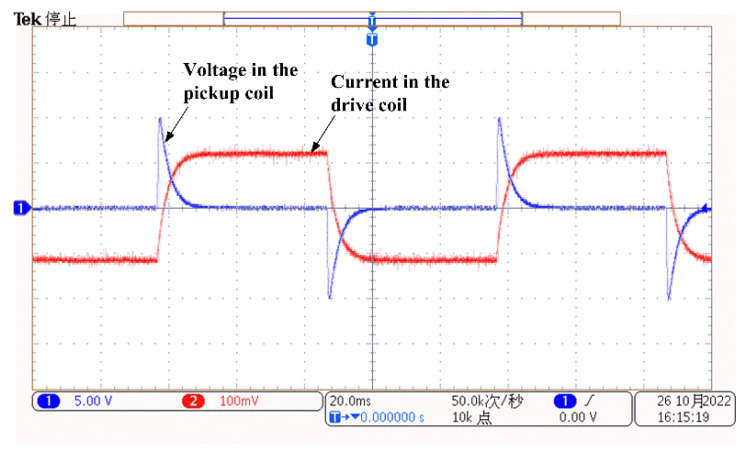
PECT probe’s driving and pickup signals, monitored using an oscilloscope.

Figure 11 shows the driving and pickup signals of the probe coils monitored by an oscilloscope (MDO3012, Tektronix, Tokyo, Japan). The current in the drive coil was observed by measuring the voltage across a 0.1 ohm sampling resistor which is in a series connection with the drive coil. The current waveform has exponential edges, although the applied voltage is a square wave, which reflects the coil inductance’s property in order to keep current lags behind the voltage (the drive coil has an inductance of 4.4 mH). According to Ohm’s law, the reading of the driving current is about 1.2 amps. The voltage in the pickup coil exhibits a pulse at each edge of the driving current, which agrees with the typical PECT signal of ferromagnetic materials.

**Figure 12:**
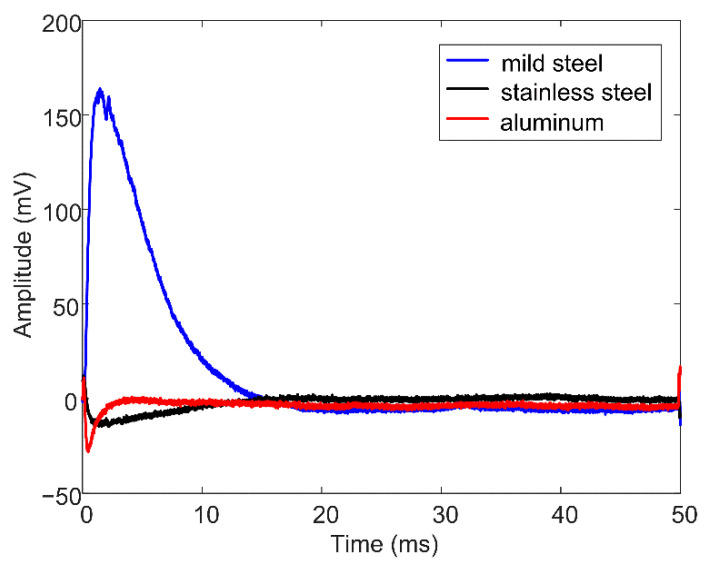
Experimental results for metal mesh made of mild steel, stainless steel, and aluminum, respectively.

Figure 12 shows the experimental results acquired from the defect-free zone covered by a metal mesh of Q195 mild steel, 304 stainless steel, and aluminum, respectively. In comparison with Figure 3, it is clear that the experiment and simulation results are consistent. The phenomenon in which only the mild steel mesh has a strong impact on the PECT signal is validated.

**Figure 13:**
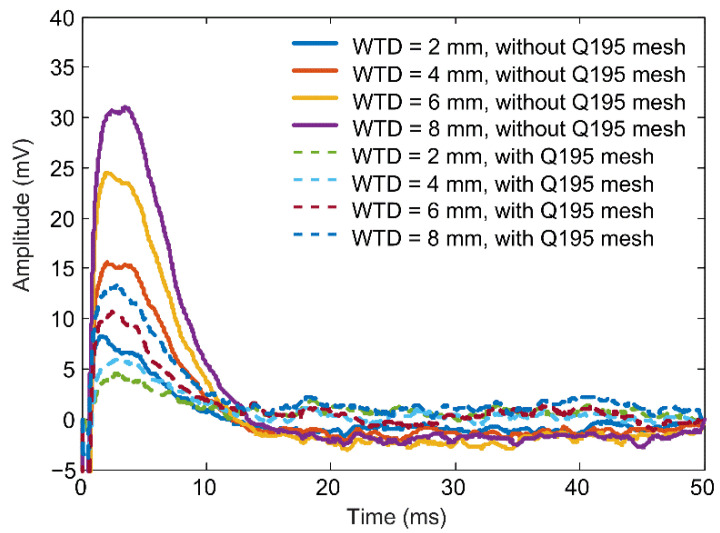
Experimental differential signals acquired from 2 mm, 4 mm, 6 mm, and 8 mm deep wall-thinning zones, without and with the covering of a Q195 mesh, respectively.

**Figure 14:**
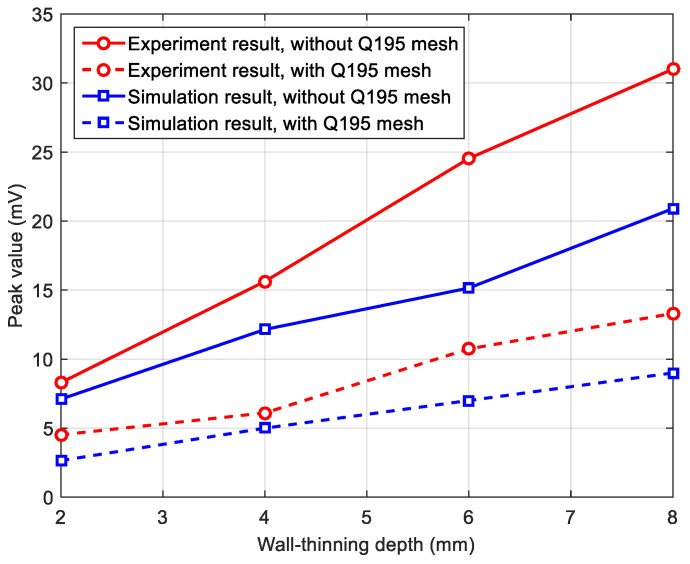
Comparison of the variations between the PECT experiment and the simulation signal peaks against the wall-thinning depth.

Figure 13 presents the experiment signals acquired from the wall-thinning sites of the sample plate, with and without the covering of Q195 mesh. The signal from the defect-free zone was used as the reference signal, and the difference process was then applied to the measured 2 mm, 4 mm, 6 mm and 8 mm wall-thinning signals. The signal curves have single peaks in the early phase. As predicted by the simulation, the signal amplitude increases regularly with the increase in the wall-thinning depth (WTD), and the signals obtained with a Q195 mesh laid in the insulation are always weaker than those obtained without a Q195 mesh. Figure 14 plots the variations in signal peak value against the wall-thinning depth. For comparison, the simulation results are also presented. As in the experiment, the signal from the pickup coil was amplified by the pre-amplifier, while in the simulation, the signal is directly extracted, and the simulation signal peaks are multiplied by a factor of 50 (the gain of the pre-amplifier) so that they are comparable to the experimental signal peaks. It is noted that the simulation results are smaller than the experiment results. The discrepancy is probably due to the quality of the handmade probe coils and the deviation of the conductivity and permeability values used in the simulation from the actual properties of the Q235 and Q195 carbon steels. In spite of this, the figure confirms that installation of a mild steel mesh in the insulation causes decreases in both the PECT signal amplitude and sensitivity (the change rate of the peak value to the thinning depth) when using the early-phase peak value as the signal feature to evaluate CUI.

## 4. Discussions and Conclusions

### 4.1. Discussions

As a follow-up study of Ref. [2], this work focused on the surface distribution of eddy currents at the time of the turning off of the driving voltage, rather than examining the overall process of eddy current diffusion. The calculated probe footprint was smaller than the one calculated based on the cross-sectional distribution of eddy currents at the characteristic time. This is easy to explain, since the eddy current is induced on the surface in the beginning and then starts a simultaneous downward and outward diffusion into the material.

Given that corrosion under insulation (CUI) happens on the outside of the insulated object, the corrosion depth was approximated as an increment of the probe lift-off, which mainly influences the early-phase signal. Since the early-phase signal feature emerges in the very beginning of the signal, the use of a low-frequency excitation to make the eddy current fully penetrate through the specimen becomes unnecessary. Therefore, the excitation frequency can be increased significantly to improve the inspection efficiency. Meanwhile, in contrast to the complicated linear fitting to calculate the decay rate, the early-phase signal has a better signal-to-noise ratio, which makes the feature extraction easier to implement, as well as more reliable, and also reduces the need for hardware in signal postprocessing.

Another point that differs from our previous work lies in the excitation mode of the probe. There are generally two excitation modes in PECT inspection, i.e., the square-wave current and the square-wave voltage. In the previous work, a square-wave current was utilized to generate a sudden shutdown of the current in the drive coil, although the shutdown was not ideally stepped because of the coil’s inductance effect. The abruption of excitation current induces a burst of eddy current in the sample plate, marking the beginning of eddy-current diffusion, but also resulting in a high voltage spike (up to tens to hundreds of volts) in the pickup coil. Hence, the acquired early-phase voltage signal was cut by the preamplifier. To exploit the early-phase signal, the amplifier saturation must be addressed. In the present work, a square-wave voltage was applied to the drive coil. By this means, the excitation current was exponentially varied at the edges of the applied voltage. The exponential change is slower and gentler than the sudden shutdown, which makes the induced eddy current relatively weaker in the beginning. Therefore, the voltage sensed in the pickup coil can always be kept below the threshold of amplifier saturation, providing a prerequisite for analyzing the early-phase signal.

### 4.2. Conclusions

This work studied the effect of the supporting metal mesh on the PECT probe footprint and the early-phase signal feature for the inspection and evaluation of CUI. The peak value, an early-phase signal feature, was found to be well correlated with the corrosion depth. Simulation and experimental results revealed that the installation of a mild steel mesh in the insulation could result in enlargement of the probe footprint, weakening of the pickup signal, and a decrease in the change rate of the peak value to corrosion depth. The findings are consistent with the results previously reported in [2], in which the decay rate, a late-phase signal feature, was employed to evaluate the remaining thickness due to CUI.

In practical cases, the insulation thickness might be not uniform due to installation error, poor maintenance, or gravity settling, which means that the probe lift-off itself is variable and therefore, renders the use of a lift-off-related early-phase signal feature invalid. Under such circumstances, it is recommended to use the late-phase signal features, such as the decay rate, as they are immune to the lift-off variation. Nonetheless, the presented method for CUI evaluation can be effective when the insulation has a fixed and uniform thickness throughout its entire service life, for example, in the cases of the concrete fireproofing of sphere legs and vessel skirts. Another applicable scenario is the steel-jacketed steel insulation pipes and the double-walled tanks, where the distance between the casing and the inner component is constant [23]. Therefore, this work also provides an alternative for evaluating CUI in these particular situations. The compensation and reduction of the effect caused by the metal mesh will be addressed in the near future.

## Figures and Tables

**Table 1:** Metal mesh parameters.

Parameter	Value
Conductivity (MS/m)	1.35, 2, 10, 21.6
Relative magnetic permeability	1, 100, 200, 300, 400, 500
Wire diameter (mm)	1, 1.5, 2, 2.5, 3
Hole side length (mm)	10, 16, 24, 30
Position (mm)	10, 20, 30, 40, 50

**Table 2:** Probe coil parameters.

Parameter	Drive Coil	Pickup Coil
Inner diameter (mm)	20	8
Outer diameter (mm)	60	18
Height (mm)	30	15
No. of turns	500	350
Wire diameter (mm)	1	0.4

## Data Availability

The data supporting reported results by the authors can be sent by e-mail.
